# Ethyl acetate extract of *Knoxia roxburghii* (Rubiaceae) down-regulates ECHDC1, CAMK2D, DDB1, UBA6, BIRC6, and HK1 proteins and ameliorates the symptoms of diabetes mellitus

**DOI:** 10.3389/fphar.2025.1587858

**Published:** 2025-07-22

**Authors:** Xinge Wang, Xiaoqiao Tian, Yang Xu, Rong Li, Gusha Qumo, Jingping Li, Niman Bao, Maoru Li, Bin Qiu

**Affiliations:** ^1^ School of Traditional Chinese Medicine, Yunnan University of Chinese Medicine, Kunming, Yunnan, China; ^2^ Yunnan Baiyao Group Wuxi Pharmaceutical Co., Ltd., Wuxi Jiangsu, China; ^3^ College of Ethnic Medicine, Yunnan University of Chinese Medicine, Kunming, Yunnan, China

**Keywords:** proteomics, metabolomics, diabetes, herb extract, PRM, oxidative

## Abstract

**Objective:**

To evaluate the effects of *Knoxia roxburghii* on blood glucose levels in diabetic rats and to investigate its underlying mechanisms of action using proteomics and metabolomics.

**Methods:**

Streptozotocin (STZ)-induced diabetic rats were treated with different doses of *K. roxburghii* extract. Proteomics and metabolomics analyses were performed using pancreatic proteins and serum samples, and the proteomics findings were validated via parallel reaction monitoring (PRM).

**Results:**

Compared with the model group, rats in the treatment group showed improved diabetic symptoms. Fasting blood glucose (FBG), glycated serum protein (GSP), pancreatic malondialdehyde (MDA), and the area under the curve of oral glucose tolerance test (OGTT-AUC) were significantly decreased (*P* < 0.01, *P* < 0.05), while superoxide dismutase (SOD), homeostasis model assessment of β-cell function (HOMA-β), and fasting insulin (FINS) were significantly increased (*P* < 0.01, *P* < 0.05). Histological analysis revealed an increased pancreatic islet cell area in the treatment group. Proteomic analysis identified six significantly downregulated proteins validated by PRM: Ethylmalonyl-CoA Decarboxylase 1 (ECHDC1), Calcium - Dependent Protein Kinase II Delta (CAMK2D), DNA Damage - Binding Protein 1 (DDB1), Ubiquitin-Like Modifier-Activating Enzyme 6(UBA6), Baculoviral IAP Repeat - Containing Protein6(BIRC6), and Hexokinase 1(HK1). These proteins were associated with six key metabolic pathways, including butyric acid metabolism, propionic acid metabolism, and the mTOR signaling pathway.

**Conclusion:**

The ethyl acetate extract of *K. roxburghii* reduces endogenous glucose production by inhibiting gluconeogenesis, alleviates oxidative stress in pancreatic cells, and preserves pancreatic islet architecture. These effects contribute to increased insulin secretion, improved glycemic control, and alleviation of diabetic symptoms in STZ-induced rats. These findings not only provide mechanistic insights into the ethnopharmacological basis for the traditional use of *K. roxburghii* in diabetes management, but also establish a scientific rationale supporting its clinical application through the regulation of hepatic gluconeogenesis and pancreatic β-cell preservation.

## 1 Introduction

Diabetes is a metabolic disease characterized by hyperglycemia, primarily caused by defective insulin secretion or impaired insulin action. It is classified into type I and type II diabetes, with type II diabetes being a glucose metabolism disorder resulting from insulin resistance, accounting for approximately 90% of all diabetes cases. In 2021, an estimated 537 million individuals were living with diabetes, with projections suggesting an increase to 643 million by 2030 and 783 million by 2045 (Magliano et al., 2021, Boyko and IDF Diabetes Atlas 10th edition scientific committee 2021). Furthermore, in 2021, 541 million individuals were estimated to have impaired glucose tolerance, and over 6.7 million people aged 20–79 died due to diabetes-related complications. As a chronic metabolic disorder, diabetes requires continuous medication for management rather than offering a cure, driving ongoing research into more effective treatments. In addition to conventional hypoglycemic drugs such as metformin and rosiglitazone, the discovery of natural anti-diabetic compounds like artemisinin has broadened treatment options. The pharmacology and active components of traditional Chinese medicines (TCMs) are now focal points in diabetes research.

TCM is commonly used in the clinical management of diabetes in China. Studies have demonstrated that various natural components in TCMs, including polysaccharides, polyphenols, anthraquinones, alkaloids, saponins, and flavonoids, can effectively alleviate diabetic symptoms and reduce the risk of complications. Among these, anthraquinones, including emodin, rhein, lignans, and quercetin, have been shown to significantly improve diabetic symptoms (Chen et al., 2023a; Liu et al., 2024; Song, 2018; Lin and Zhang, 2023). As a result, TCMs, known for their minimal side effects and rich bioactive compounds, play an increasingly important role in diabetes treatment strategies. Therefore, the search for novel herbal medicines and natural compounds for diabetes prevention and treatment remains a key area of research.

*K. roxburghii* is derived from the dried root of *K. roxburghii* (Spreng.), Rubiaceae. According to the 2020 edition of the Chinese Pharmacopoeia, it is traditionally used to treat conditions such as edema, fluid accumulation in the chest and abdomen, phlegm, retrograde cough, asthma, adverse bowel movements, carbuncles, swelling, and sores (C. Liu et al., 2024; Zhang et al., 2023b). In TCMs, *K. roxburghii* has long been used in the folk treatment of diabetes mellitus, demonstrating significant clinical efficacy. However, due to the harsh environmental conditions required for its growth and its limited reproductive capacity, *K. roxburghii* has become a rare and endangered medicinal herb. Furthermore, the chemical composition and pharmacological effects of *K. roxburghii* remain insufficiently explored. Modern medical research lacks comprehensive documentation and reports on its potential to treat diabetes mellitus and its blood glucose-lowering effects. Research indicates that *K. roxburghii* is rich in anthraquinones, which are its primary natural compounds. To date, 51 compounds, including 3-hydroxycorbazone and red halberdine, have been identified as major anthraquinones and their derivatives (Pu, 2024). These natural compounds contribute to *K. roxburghii*’s diverse pharmacological properties. Current research focuses mainly on its laxative effects, with recent studies highlighting its bacteriostatic effects and potential antitumor activity (Chen et al., 2023b; Chen et al., 2022; Qin et al., 2013; Yang, 2013). Moreover, anthraquinone compounds in *K. roxburghii* have been shown to inhibit diabetes-related PTP1B protein activity and the production of advanced glycation end products (AGEs) (Yoo et al., 2010; Zhang et al., 2022). Building on the current understanding of *K. roxburghii*’s composition and its potential relevance to diabetes treatment, this study used artificially cultivated *K. roxburghii* in animal experiments to further explore its hypoglycemic properties and effects on diabetes.

Genomics provides an efficient means of systematically analyzing biological functions and mechanisms through high-throughput sequencing, ultra-resolution mass spectrometry, and advanced biochemical analyses. Since its introduction in 1986, genomics has driven the rapid development of related technologies, including transcriptomics, proteomics, and metabolomics (Williamson et al., 2023). These tools have been successfully applied to diabetes research. They reveal biomarkers, glucose responses in insulin secretion, and molecular features of the disease (Doumatey et al., 2024; Li et al., 2015; Yang, 2013; Liu et al., 2023).

Building on this foundation, the present study constructed a diabetes model using Sprague-Dawley (SD) rats. After pharmacological intervention, pancreatic tissues obtained post-mortem were analyzed using proteomic analysis, while serum from the abdominal aorta was examined via metabolomics. This approach aimed to pharmacodynamically evaluate the hypoglycemic effects of *K. roxburghii* and investigate its mechanisms of action. Moreover, this research provides a reference for the pharmacological activity of *K. roxburghii* and the development of hypoglycemic drugs.

## 2 Materials and methods

### 2.1 Reagents and instruments

The following reagents and instruments were used: streptozotocin (STZ, S0130, Sigma-Aldrich, CA, United States), rosiglitazone tablets (H20030569, Hengrui, Chengdu, China), 0.1 M sodium citrate buffer (C1013, Solarbio, Beijing, China), glucometer (GA-3, Sinocare, Changsha, China), sodium pentobarbital (BC1040, Luton Biotechnology Co., Ltd., Beijing, china), rat insulin (INS) ELISA kit (PA03X20Z6153, Elabscience, Wuhan, China), glycated serum protein assay kit (GSP, A037-2-1, Jiancheng, Nanjing, China), superoxide dismutase assay kit (SOD, A001-3, Jiancheng, Nanjing, China), malondialdehyde assay kit (MDA, A003-1, Jiancheng, Nanjing, China), and total protein quantitative assay kit (TP, A045-4, Jiancheng, Nanjing, China). Organic reagents were purchased from Tianjin ZhiYuan Reagent (Tianjin, China).

### 2.2 Preparation of ethyl acetate extract of *Knoxia roxburghii*

*Knoxia roxburghii* was obtained from Dali Yuansheng Agricultural Technology Co., Ltd. (Yunnan Province, China), derived specifically from the cultivated and selectively bred dried roots of *K. roxburghii*. This cultivar, designated ‘Yunji 2′, has been registered as a new horticultural plant variety by the Yunnan Provincial Forestry and Grassland Administration (Registration Number: 20230016). The dried, impurity-free *K. roxburghii* was powdered and soaked in 10 times its weight of 75% ethanol for 7 days (1 kg *K roxburghii*: 10 L of 75% ethanol). The filtrate was concentrated to a suitable volume, and the residue was soaked in 8 times its weight of 75% ethanol for 5 days (1 kg *K roxburghii*: 8 L of 75% ethanol). The filtrate was then concentrated and mixed with three times their volume of water, left to stand for 48 h, and then filtered to obtain the *K. roxburghii* (KR) extract. The extract was further concentrated and dissolved in the same volume of pure water. Sequential extractions were performed with ethyl acetate, n-butanol, and chloroform (each three times the extract volume). The ethyl acetate phase was collected, concentrated, and evaporated to remove the solvent. The resulting odorless extract was freeze-dried and stored at −20 °C. The extraction yield of the ethyl acetate fraction (KR-EA) was 0.27% under the specified conditions.

### 2.3 Animals and induction of type 2 diabetes

Male Sprague-Dawley rats were purchased from Beijing Vital River Laboratory Animal Technology and housed at the Experimental Animal Center of Yunnan University of Traditional Chinese Medicine under controlled conditions (22°C ± 2 C, 55% ± 5% humidity). Experimental procedures complied with “The Regulations on the Administration of Experimental Animals” of the People’s Republic of China’s State Science and Technology Commission. The animal study was approved by Yunnan University of Traditional Chinese Medicine (D-062023023). Rats were allowed to acclimate to the laboratory environment for 7 days before experimentation. Except for the normal group of rats, diabetes was induced by intraperitoneal injection of STZ at a dose of 40 mg/kg, dissolved in 0.05 M citrate buffer (pH 4.5) (Jin S.et al., 2022). Three days later, fasting blood glucose (FBG) was measured from tail vein blood using a glucometer. Rats with FBG ≤11.1 mM/L received a supplemental STZ dose of 30 mg/kg. After 3 days, rats with FBG ≥11.1 mM/L were considered successfully modeled for diabetes. Diabetic rats were divided into six groups based on blood glucose levels: normal control (NC), model group (MOD), rosiglitazone group (RG, 0.38 mg/kg), low-dose KR ethyl acetate extract group (KR-EA-L, 0.3 mg/kg), medium-dose KR ethyl acetate extract group (KR-EA-M, 0.6 mg/kg), and high-dose KR ethyl acetate extract group (KR-EA-H, 1.2 mg/kg). The NC and MOD groups received pure water containing 0.1% DMSO. The entire experiment was conducted using oral administration, and the dosing period lasted for 35 days. Rats were fasted overnight with free access to water prior to dissection. Euthanasia was performed via intraperitoneal injection of pentobarbital solution (150 mg/kg), and blood samples were collected through transabdominal aortic puncture (Kollias et al., 2023).

### 2.4 Glucose and oral glucose tolerance test

Blood glucose levels and body weight of the rats in each group were measured every 5 days. The rats were fasted starting at 8:00 a.m., and blood glucose levels were measured 3 h later using a glucometer. Blood was collected from the tail vein, with the first drop wiped off, before measuring the second drop. Data were recorded for each measurement. For the oral glucose tolerance test (OGTT), animals were fasted for 12 h and given glucose dissolved in water (3 g/kg body weight) by gavage. Blood samples were collected from the tail vein at 0, 30, 60, 90, and 120 min to measure blood glucose concentrations. The area under the curve (AUC) for the blood glucose time profile was calculated using the trapezoidal rule with the following formula:

$$\text{AUC}=0.5\times \left({BG}_{o}\;\min+{BG}_{30}\;\min\right)+0.5\times \left({BG}_{30}\;\min+{BG}_{60}\;\min\right)+0.5\times \left({BG}_{60}\min+{BG}_{90}\min\right)+0.5\times \left({BG}_{90}\;\min+{BG}_{120}\min\right),$$

Where BG represents the blood glucose concentration at the respective time points.

### 2.5 Hematoxylin and eosin (H&E) staining

Pancreatic tissues were fixed in 4% paraformaldehyde at 4°C for 24 h. The samples were sequentially embedded in paraffin, sectioned, and stained with H&E. Following dehydration and mounting, histopathological changes were observed under an optical microscope.

### 2.6 Proteomic analyses

Sample lysis and protein extraction were conducted using SDT buffer (4% SDS, 100 mM Tris-HCl, 1 mM DTT, pH 7.6). Protein quantification was performed using the BCA Protein Assay Kit. Proteins were digested using the filter-assisted sample preparation (FASP) method described by Matthias Mann. Digested peptides were desalted using C18 cartridges (Empore™ SPE Cartridges C18, standard density, I.D. 7 mm, volume 3 mL, Sigma), concentrated via vacuum centrifugation, and reconstituted in 40 µL of 0.1% (v/v) formic acid.

For each sample, 200 µg of protein was mixed with 30 µL of SDT buffer (4% SDS, 100 mM DTT, 150 mM Tris-HCl, pH 8). Low molecular weight components, such as detergents and DTT, were removed through repeated ultrafiltration (Microcon unit, 10 kD) with UA buffer (8 M urea, 150 mM Tris-HCl, pH 8.0). Then, 100 μL of iodoacetamide (100 mM in UA buffer) was added to block reduced cysteine residues, followed by incubation in the dark for 30 min. The membrane was washed three times with 100 µL of UA buffer and twice with 100 µL of 25 mM ammonium bicarbonate buffer. Proteins were digested overnight at 37°C with 4 µg of trypsin (Promega) in 40 µL of 25 mM ammonium bicarbonate buffer, and peptides were collected as filtrates. Peptides were desalted using C18 cartridges, vacuum-concentrated, and reconstituted in 40 µL of 0.1% (v/v) formic acid. Peptide content was estimated based on the extinction coefficient of the solution (0.1 g/L) using UV spectral density at 280 nm, considering the frequency of tryptophan and tyrosine residues in vertebrate proteins. Liquid chromatography-tandem mass spectrometry (LC-MS/MS) analysis was performed using a timsTOF Pro mass spectrometer (Bruker) coupled to a NanoElute system (Bruker Daltonics). Samples were analyzed over run times of 60, 120, and 240 min to acquire comprehensive proteomic data.

### 2.7 Metabolomics

Fasting blood samples were collected in 5 mL Vacutainer tubes containing ethylenediaminetetraacetic acid (EDTA) as a chelating agent. The samples were centrifuged at 14,000 × *g* for 15 min at 4°C, and the supernatant was collected. Plasma samples (150 µL each) were stored at −80°C until further analysis. Before analysis, the samples were thawed at 4 °C. Each sample (100 µL) was mixed with 400 µL of cold methanol/acetonitrile (1:1, v/v) to precipitate proteins. The mixture was centrifuged for 20 min (14,000 × *g*, 4°C), and the supernatant was dried in a vacuum centrifuge. For LC-MS analysis, the samples were reconstituted in 100 µL of acetonitrile/water (1:1, v/v), centrifuged at 14,000 × *g* for 15 min at 4°C, and the supernatant was injected into the system. QC samples are made from a mixture of samples to be tested and are tested on the machine before, during and after the LC-MS/MS injection of the samples to be tested.

The analysis was conducted using an ultra-high-performance liquid chromatography (UHPLC) system (1290 Infinity LC, Agilent Technologies) coupled to a quadrupole time-of-flight mass spectrometer (AB Sciex TripleTOF 6600). Hydrophilic interaction liquid chromatography (HILIC) separations were performed on a 2.1 × 100 mm ACQUITY UPLC BEH Amide column (1.7 µm, Waters, Ireland). In both positive and negative electrospray ionization (ESI) modes, the mobile phases consisted of 25 mM ammonium acetate and 25 mM ammonium hydroxide in water (Phase A) and acetonitrile (Phase B). The gradient program included 95% B for 0.5 min, a linear decrease to 65% B over 6.5 min, a decrease to 40% B within 1 min held for 1 min, and an increase to 95% B within 0.1 min, followed by a re-equilibration period of 3 min. MS settings included Gas1 and Gas2 set at 60, curtain gas (CUR) at 30, ISV ± F at 60, and a source voltage of ±5,500 V. For MS-only acquisition, the instrument operated in the range of *m/z* 60–1,000 Da, with a cumulative TOF-MS scan time of 0.20 s per spectrum. In automated MS/MS acquisition, the instrument operated in the range of *m/z* 25–1,000 Da, with a product ion scan accumulation time of 0.05 s per spectrum. Product ion scans were acquired using the information-dependent acquisition (IDA) method in high-sensitivity mode. The collision energy was fixed at 35 V (±15 eV), and the declustering potential was set to 60 V in positive mode and −60 V in negative mode. Isotopes within 4 Da were excluded, and up to 10 candidate ions were monitored per cycle.

### 2.8 Bioinformatic analyses

For proteomics, raw data from 12 samples were analyzed using MaxQuant 1.5.3.17 software for protein identification and quantitative analysis. Significant differentially expressed proteins (DEPs) were identified based on fold change (FC) ≥ 1.2 and *P* < 0.05. Hierarchical clustering of DEPs was performed using Cluster 3.0 and visualized with Java Treeview software. Subcellular localization predictions were conducted using the SVM-based CELLO classification system. Protein sequences of DEPs were analyzed locally for homologous sequences using NCBI BLAST^+^ client software (version 2.2.28, (National Center for Biotechnology Information, United States) and InterProScan (European Bioinformatics Institute, UK). Gene Ontology (GO) annotations were assigned using Blast2GO software (version 2.5.0), and DEPs were matched to Kyoto Encyclopedia of Genes and Genomes (KEGG) orthologies for pathway mapping.

For metabolomics, raw data were converted into MzXML files using ProteoWizard MSConvert before analysis with the XCMS software (version 3.0.6428) suite. Metabolite structures were identified by comparing accurate *m/z* values (<10 ppm), MS/MS spectra, retention times, molecular weights (with a mass error of 25 ppm), secondary fragmentation spectra, and collision energies against a local in-house database (Shanghai Applied Protein Technology). Identification results were verified to ensure identification confidence of Level 2 or above.

Following data acquisition, metabolomic datasets underwent total peak intensity normalization and SVR correction. Multivariate statistical normalization was then performed using Par (Pareto scaling). Multivariate statistical analyses, including Pareto-scaled principal component analysis (PCA) and orthogonal partial least squares discriminant analysis (OPLS-DA), were performed on the identified metabolites. Variable importance for projection (VIP) values in the OPLS-DA model was calculated to assess the contribution of each variable to group classification. The significance between the two independent groups was determined using Student’s t-test. Metabolites with significant changes (VIP >1, *P* < 0.05, and FC ≥ 1.2) were selected for subsequent correlation analysis.

### 2.9 Parallel reaction monitoring (PRM)

Parallel Reaction Monitoring (PRM) is a high-resolution, high-precision mass spectrometry-based ion monitoring technology that selectively detects and quantifies target proteins and peptides (e.g., peptides undergoing post-translational modifications). Based on the proteomics results, Peptide information suitable for PRM analysis was imported into Xcalibur software (Thermo Fisher Scientific, United States) for PRM method setup. Approximately 1 µg of peptide from each sample was mixed with 20 fmol of standard peptide (PRTC: ELGQSGVDTYLQTK) for detection.

Chromatographic conditions included the use of 0.1% formic acid in water as liquid A and 0.1% formic acid in 84% acetonitrile aqueous solution as liquid B. The column was equilibrated with 95% liquid A. Gradient elution was performed as follows: 0–2 min, a linear gradient of liquid B from 5% to 10%; 2–45 min, 10%–30%; 45–55 min, 30%–100%; and 55–60 min, liquid B maintained at 100%. MS conditions involved positive ion detection mode, with a total analysis time of 60 min. The primary MS scan range was *m/z* 300–1,800, with an MS resolution of 60,000 (*m/z* 200), an AGC target of 3 × 10^6^, and a maximum injection time (max IT) of 200 m. 20 PRM scans for target proteins were acquired following each primary MS scan using an isolation window of 1.6 Th, an MS resolution of 30,000 (*m/z* 200), an AGC target of 3 × 10^6^, and a max IT of 120 ms. The MS2 start type was HCD, with a normalized collision energy of 27 eV.

Skyline (version 3.5.0, Skyline Software Systems, Inc.) was used to analyze the PRM raw files. Three sub-ions with the highest and most consecutive peptide abundance were selected for quantitative analysis for each target peptide. The peak area of each target peptide was exported from Skyline, and the raw values were corrected using the heavy isotope labeling of the internal standard peptide. This correction provided the relative expression levels of peptides across samples. The average relative expression of the target peptide in each group was calculated and statistically analyzed. Relative expression differences of target proteins between groups were determined based on the relative expression of the corresponding peptide of each target protein in different sample groups.

### 2.10 Data analyses

Statistical analyses were conducted to assess differences between groups. Comparisons between two groups were performed using unpaired *t*-tests, while comparisons among multiple groups were analyzed using one-way analysis of variance (ANOVA). Graphical and statistical analyses were conducted using GraphPad Prism 8.4.0 (GraphPad Software, United States), SPSS 21.0 (IBM Corporation, United States)), and image analysis was conducted using ImageJ software (National Institutes of Health, United States).

## 3 Results

### 3.1 KR-EA reduces blood glucose and improves glucose tolerance in diabetic rats

**FIGURE 1:**
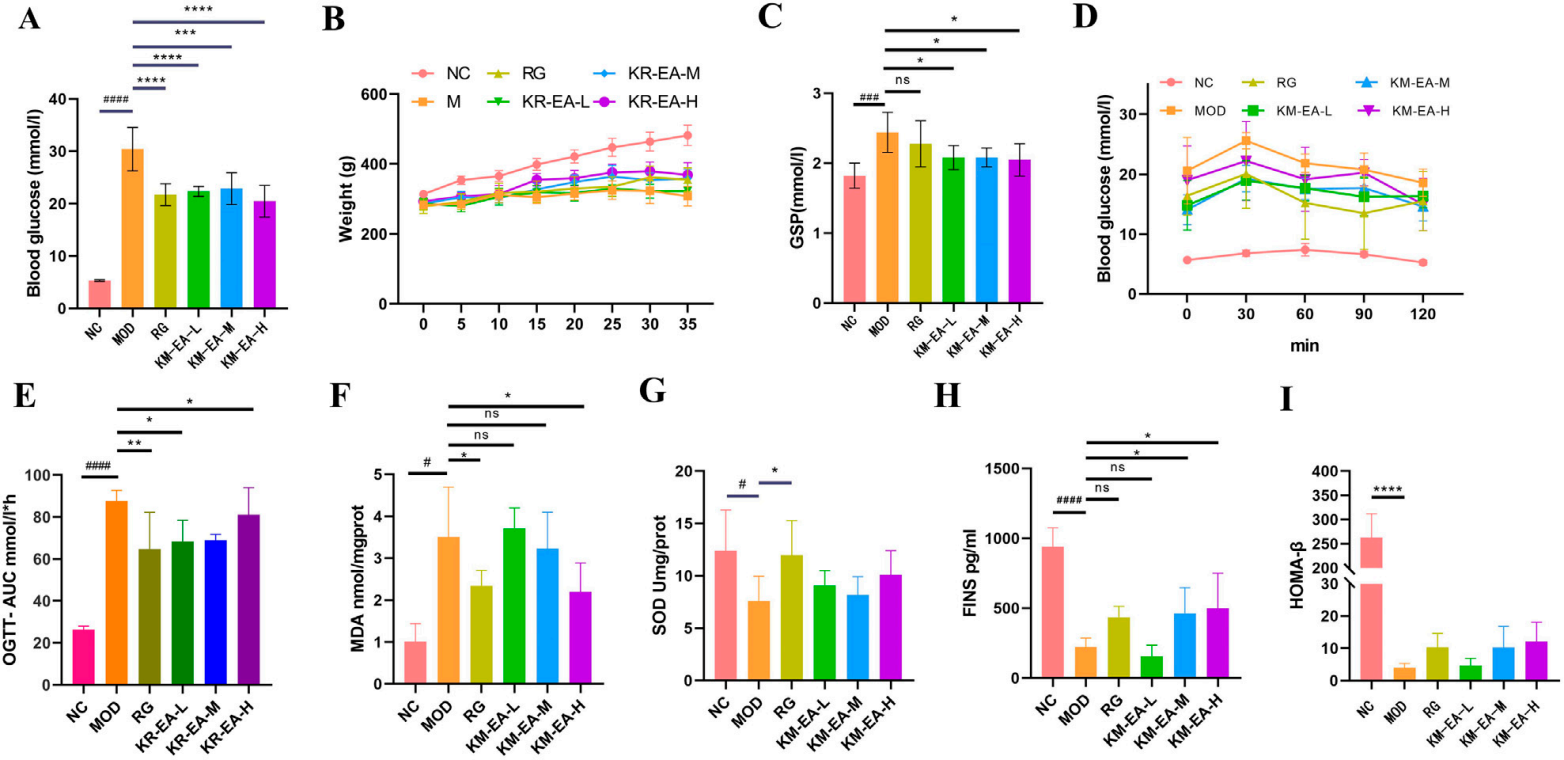
Blood glucose level of rats on the last day of drug administration. **(A)** Changes in body weight. **(B)** Glycosylated Serum Proteins (GSP)levels. **(C)** Oral Glucose Tolerance Test (OGTT) changes. **(D)** Area Under the Curve of Oral Glucose Tolerance Test (AUC of OGTT) levels. **(E)** Malondialdehyde (MDA) levels. **(F)** Superoxide Dismutase (SOD) levels. **(G)** Fasting Insulin (FINS) levels levels. **(H)** and Homeostasis Model Assessment of β-cell function (HOMA-β) levels. **(I)** in each group of rats. Data were shown as mean ± SD (n = 6), ^#^P < 0.05, ^##^P < 0.01 ,^###^P < 0.001, ^####^P < 0.0001 vs. NC group, *P < 0.05, **P < 0.01 ,***P < 0.001, ****P < 0.0001 vs. MOD group.

The hypoglycemic effect of KR-EA in diabetic rats was assessed by measuring FBG every 5 days and performing an OGTT on the day before the final administration. As shown in Figure 1A, the FBG levels of rats in all three KR-EA dose groups were significantly reduced compared with the MOD group (*P* < 0.05). Among the KR-EA treatment groups, the high-dose group (KR-EA-H) had the most significant glucose-lowering effect, with FBG levels lower than those observed in the RG group. Figure 1C displays serum GSP levels on the final day of administration, which reflect average blood glucose levels over the previous 2–3 weeks. All KR-EA-treated groups showed significant reductions in serum GSP levels compared with the MOD group, which was consistent with the observed FBG trends. While rats in the NC group showed continuous weight gain, those in the MOD group experienced either stagnant or decreasing body weight. On the other hand, the body weight of rats in the KR-EA treatment groups did not decrease continuously and demonstrated a increasing trend. As quantitatively detailed in Table 1, all therapeutic groups exhibited significant reversal of diabetes-induced weight loss, with the KR-EA-H group demonstrating a body weight gain rate comparable to the rosiglitazone reference group (25.8% vs. 27.6%). Oral glucose tolerance tests (OGTT) revealed distinct metabolic recovery patterns:Rats in the KR-EA groups showed a transient increase in blood glucose, followed by a subsequent decline. The NC group maintained stable glucose tolerance, while the MOD group displayed persistently elevated blood glucose levels across all time points. Relative to the MOD group, the rate of glucose increase in the KR-EA-treated groups slowed significantly, indicating improved glucose tolerancetu Figure 1D. To comprehensively evaluate dynamic glucose metabolism in diabetic rats, we conducted standardized oral glucose tolerance test (OGTT) with subsequent area under the curve (AUC) analysis. As shown in Figure 1E, the AUC of blood glucose levels was significantly increased in the MOD group compared with the NC group (*P* < 0.001). On the other hand, the AUC was significantly reduced in the KR-EA-L, KR-EA-H, and RG groups compared with the MOD group (*P* < 0.05). These results demonstrate that KR-EA, administered by gavage, effectively reduced blood glucose levels, prevented body weight loss, and improved glucose tolerance in diabetic rats.

**TABLE 1:** The effect of *Knoxia roxburghii* extract intervention on the body weight change rate in diabetic rats.

	Day 0 Weight/g	Day 35 Weight/g	Change% ( $\bar{X}$ ± SD, n = 6)
NC	313.53	485.54	54.86% ± 0.13
MOD	285.66	302.46	5.88% ± 0.12
RG	278.60	355.47	27.60% ± 0.08
KR-EA-L	286.43	318.40	11.16% ± 0.09
KR-EA-M	286.78	345.75	20.56% ± 0.06
KR-EA-H	293.23	368.90	25.80% ± 0.12

Note: Day 0 Weight/g” refers to the body weight on the first day of administration, and “Day 35 Weight/g” refers to the body weight on the 35th day of administration, measured in grams (g), The percentage of weight change is expressed as the mean ± standard deviation. (n = 6 per group).

### 3.2 KR-EA improves pancreatic status in rats

Oxidative damage in pancreatic tissues was measured to evaluate pancreatic function in rats. MDA levels in the KR-EA-treated groups decreased with increasing doses compared with the MOD group (Figure 1F). SOD levels were higher in all KR-EA-treated groups than in the MOD group (Figure 1G). The KR-EA-H group showed a significant reduction in pancreatic MDA content (*P* < 0.05), indicating that KR-EA reduces oxidative damage in pancreatic tissues, although its protective effect did not reach the level of rosiglitazone.

Serum insulin content was also measured, revealing significantly lower insulin levels in the MOD group compared with the NC group (Figure 1H). In KR-EA-treated groups, insulin levels increased with increasing doses, with the KR-EA-H group showing the highest serum insulin content. Furthermore, the HOMA-β index, an indicator of pancreatic function, was highest in the KR-EA-H group, suggesting greater improvement in pancreatic function in this group (Figure 1I).

**FIGURE 2:**
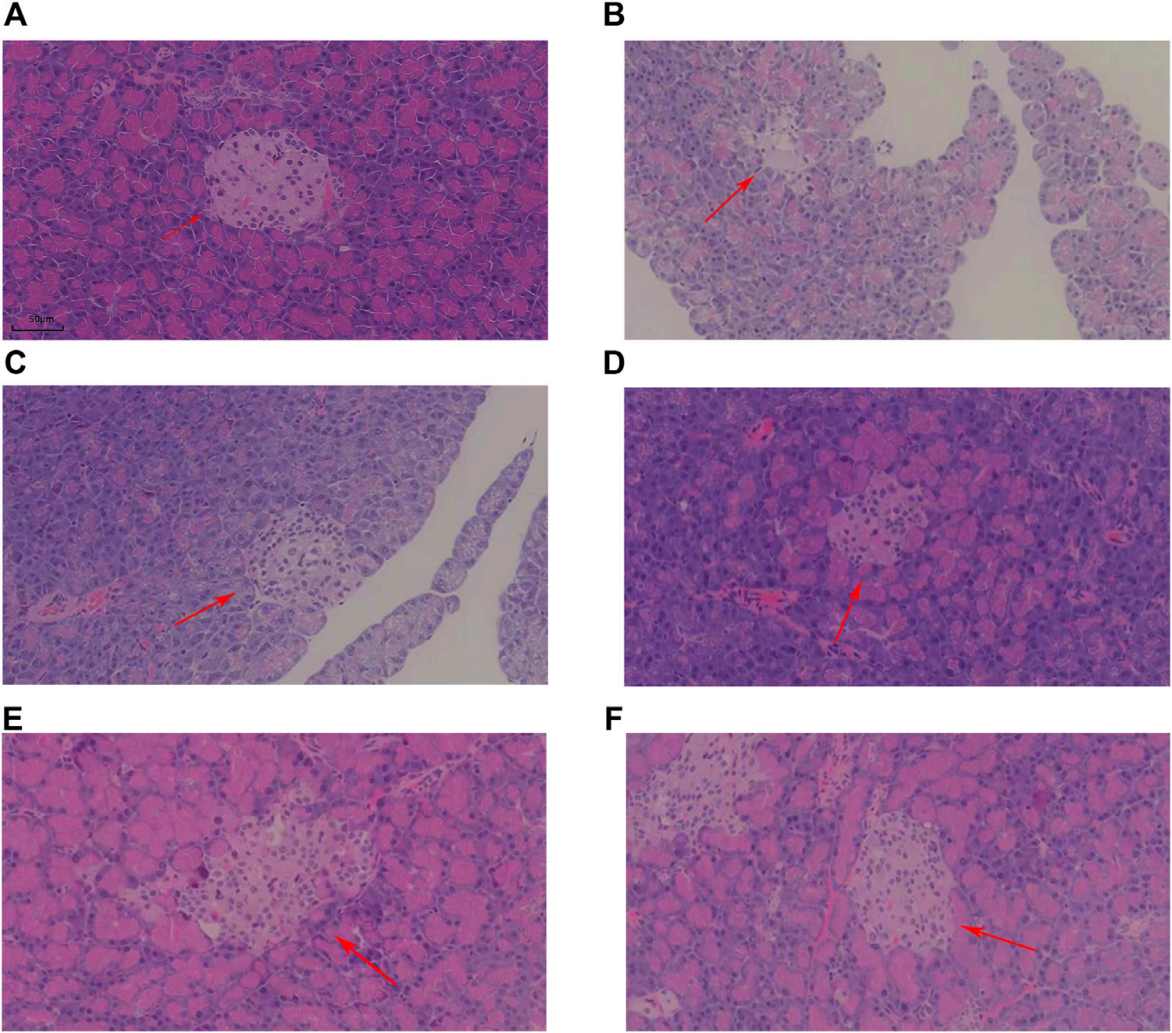
Representative hematoxylin and eosin (H&E)-stained sections of pancreatic tissue in each group. The HE section of rat pancreas in NC group **(A)**, the HE section of rat pancreas in MOD group **(B)**, the HE section of rat pancreas in RG group **(C)**, the HE section of rat pancreas in KR-EA-L group **(D)**, the HE section of rat pancreas in KR-EA-M group **(E)** and the HE section of rat pancreas in KR-EA-H group **(F)**. The arrows point to islet cells.

Histopathological evaluation was performed using a high-resolution digital slide scanning system (SQA-1000, Guangzhou Borui Biotechnology) with multispectral imaging capabilities. And photographs of pancreatic islets were taken in 4X field of view and islet area was calculated using ImageJ. Representative H&E sections of pancreatic tissue from each group are shown in Figure 2. The arrows indicate islets, which are the endocrine components of the pancreas. In the NC group, islets were flocculent, with rounded cells, clear morphology, and large size. However, islets in the MOD group displayed a disrupted structure and reduced size. Post-treatment, islet cell morphology improved across all KR-EA-treated groups, with cells in the KR-EA-H group appearing fuller and more similar to those in the NC group.

Under the 4X field of view, the pancreatic islet area varied among different experimental groups. The model group showed the lowest islet proportion (0.03%). Treated rats exhibited increased islet area proportions, with the medium-dose group (0.06% ± 0.04) demonstrating a higher proportion than the positive control group (0.05% ± 0.02) (Table 2). This indicates that KR-EA not only improved islet cell status but also promoted islet cell growth, displaying certain advantages compared to the positive control group. Furthermore, the exocrine pancreas of the NC group showed clear pink vesicles, while the MOD group showed smaller, less distinct vesicles with reduced fullness. In the KR-EA-M and KR-EA-H groups, the number and condition of vesicles improved, with vesicles appearing fuller and more distinct than in the MOD group. These results indicate that KR-EA treatment can reduce oxidative damage, improve insulin secretion, and improve both endocrine and exocrine pancreatic function in diabetic rats.

**TABLE 2:** The effect of *Knoxia roxburghii* extract on the islet area percentage in diabetic rats (×4, $\bar{X}$ ± SD, n = 3).

	Pancreatic islet area (um^2^)	Percentage (%)
NC	0.08 ± 0.04	0.05 ± 0.01
MOD	0.04 ± 0.02	0.03 ± 0.02
RG	0.07 ± 0.00	0.05 ± 0.02
KR-EA-L	0.05 ± 0.01	0.04 ± 0.00
KR-EA-M	0.09 ± 0.06	0.06 ± 0.04
KR-EA-H	0.06 ± 0.03	0.04 ± 0.02

Note: “Pancreatic islet area (um²)” refers to the actual size of the pancreatic islets in each rat, measured in square micrometers (um²), and “Percentage (%)” represents the percentage that the islet area occupies of the total pancreatic area. **×**4 indicates observation under a 4× magnification; Data are presented as Mean ± standard deviation (n = 3 per group).

### 3.3 Proteomics

#### 3.3.1 Analysis of expression differences

**FIGURE 3:**
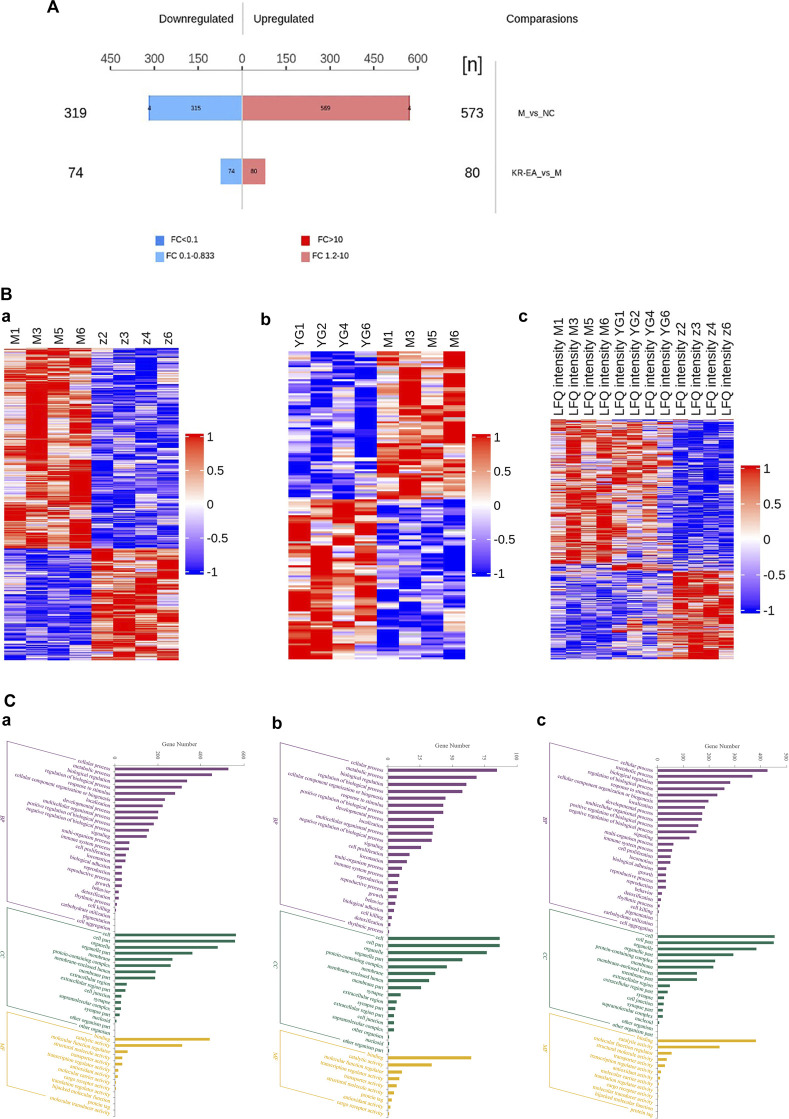
Multidimensional proteomics analysis of the therapeutic effects of *Knoxia roxburghii* on diabetes. Histogram of differentially expressed proteins between treatment groups in a diabetes study **(A)**. It highlights 319 downregulated and 569 upregulated proteins in the MOD group compared to the positive KR-EA-H group, with fold changes indicated by color. The histogram also notes 573 proteins between NC and MOD groups and 80 between KR-EA and M groups. Hierarchical clustering heat maps analyzing protein expression patterns across different experimental groups **(B)**. Hierarchical between the MOD group and the NC group **(a)**, between the KR-EA-H group and the MOD group **(b)**, and MOD/NC/KR-EA-H triple comparison **(c)**. Blue indicates low expression, white indicates medium expression, and red indicates high expression. Molecular functional enrichment histograms for three different comparisons **(C)**. Molecular functional enrichment histograms between the MOD group and the NC group **(a)**, between the KR-EA-H group and the MOD group **(b)**, and MOD/NC/KR-EA-H triple comparison **(c)**. x-coordinate is the logarithmic value of the relative quantitative values of the proteins after Log2 transformation and the y-axis listing the molecular functions. Purple indicates the highest level of enrichment significance, green indicates a moderate level of enrichment significance, and yellow indicates the lowest level of enrichment significance.

**FIGURE 4:**
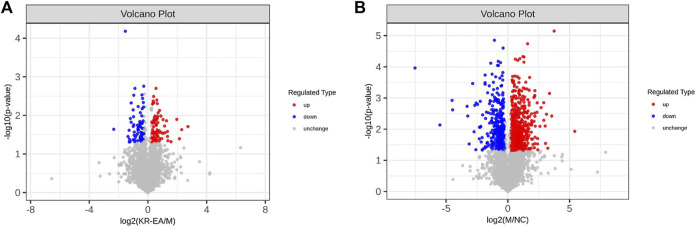
Differentially expressed proteins (DEPs) profiles based on TMT proteomics analysis. Volcano plots of DEPs between the MOD group and the positive KR-EA-H **(A)** and between the NC group and the MOD group **(B)**. x-coordinate is the logarithmic value of the relative quantitative values of the proteins after Log2 transformation, and the y-coordinate is the P-value after -log10 transformation. Red dots indicate significantly upregulated proteins and green dots indicate significantly downregulated proteins.

Protein identification in each group revealed a total of 4,589 proteins in the MOD group, 4,468 proteins in the NC group, and 4,583 proteins in the KR-EA-H group, with 4,426 overlapping proteins across the three groups. These results demonstrated greater variability between groups and good repeatability of protein identification within groups. DEPs were identified using the criteria of FC > 1.2 for upregulation, FC < 0.83 for downregulation, and *P* < 0.05 (unpaired *t*-test). A total of 892 DEPs were identified when comparing the MOD group to the NC group, including 573 significantly upregulated and 319 significantly downregulated proteins. Compared to the MOD group, 154 DEPs were identified in the KR-EA-H group, including 80 upregulated and 74 downregulated proteins (Figure 3A). The volcano plot of DEPs provides a visual representation of the number and distribution of DEPs (Figure 4). The horizontal axis represents the log2-transformed FC, while the vertical axis represents the -log10-transformed *P* value, with larger values indicating greater significance in protein expression differences.

A hierarchical clustering algorithm was applied to the DEPs to evaluate the expression patterns within and between groups, assess the rationality of the study’s grouping, and illustrate whether the changes in differential protein expression can represent the significant effects of biological treatments on the samples. The clustering results are presented as a heatmap (Figure 3B). DEPs were screened using a multiplicative change >1.2-fold and *P* < 0.05 as the criteria. The heatmap revealed a high degree of similarity among samples within groups and a low degree of similarity between groups, effectively distinguishing the comparative groups. These results indicate that the DEPs reflect the significant biological effects of the treatments on the samples.

#### 3.3.2 GO functional analysis

GO is a standardized system for the functional classification and annotation of proteins. It categorizes proteins into three main domains: biological process (BP), molecular function (MF), and cellular component (CC) (Ashburner et al., 2000). GO provides a standardized framework to describe the properties of genes and their products, assigning all proteins to secondary functional annotation levels, as illustrated in Figure 3Ca.

In the comparison of the MOD group with the NC group (Figure 3Ca), DEPs were primarily associated with BPs such as cellular processes, biological regulation, regulation of biological processes, stimulus responses, and cellular organic nitrogen compounds. The main CCs included organelles, cell membranes, and protein complexes, while MFs included catalytic activity, structural molecular activity, molecular transduction activity, and molecular functional activity. In the KR-EA groups compared with the MOD group, DEPs were primarily involved in BPs such as cellular processes, metabolic processes, bioregulatory processes, cellular organic nitrogen compounds, and stimulation responses (Figure 3Cb). The main CCs included organelles, cell membranes, and protein complexes, while MFs included catalytic activity, molecular functional activity, molecular transduction activity, and other related functions. A combined analysis of the three groups (Figure 3Cc) revealed that DEPs were commonly involved in BPs, such as cellular processes, metabolic processes, bioregulation, and stimulus responses. The primary CCs included organelles, protein complexes, and cell membranes. MFs included catalytic activity, structural molecular activity, molecular functional activity, and molecular transduction activity. These results highlight the functional diversity of DEPs and their roles in key biological processes, molecular functions, and cellular components across the treatment groups.

#### 3.3.3 Significant enrichment analysis of the KEGG pathway

**FIGURE 5:**
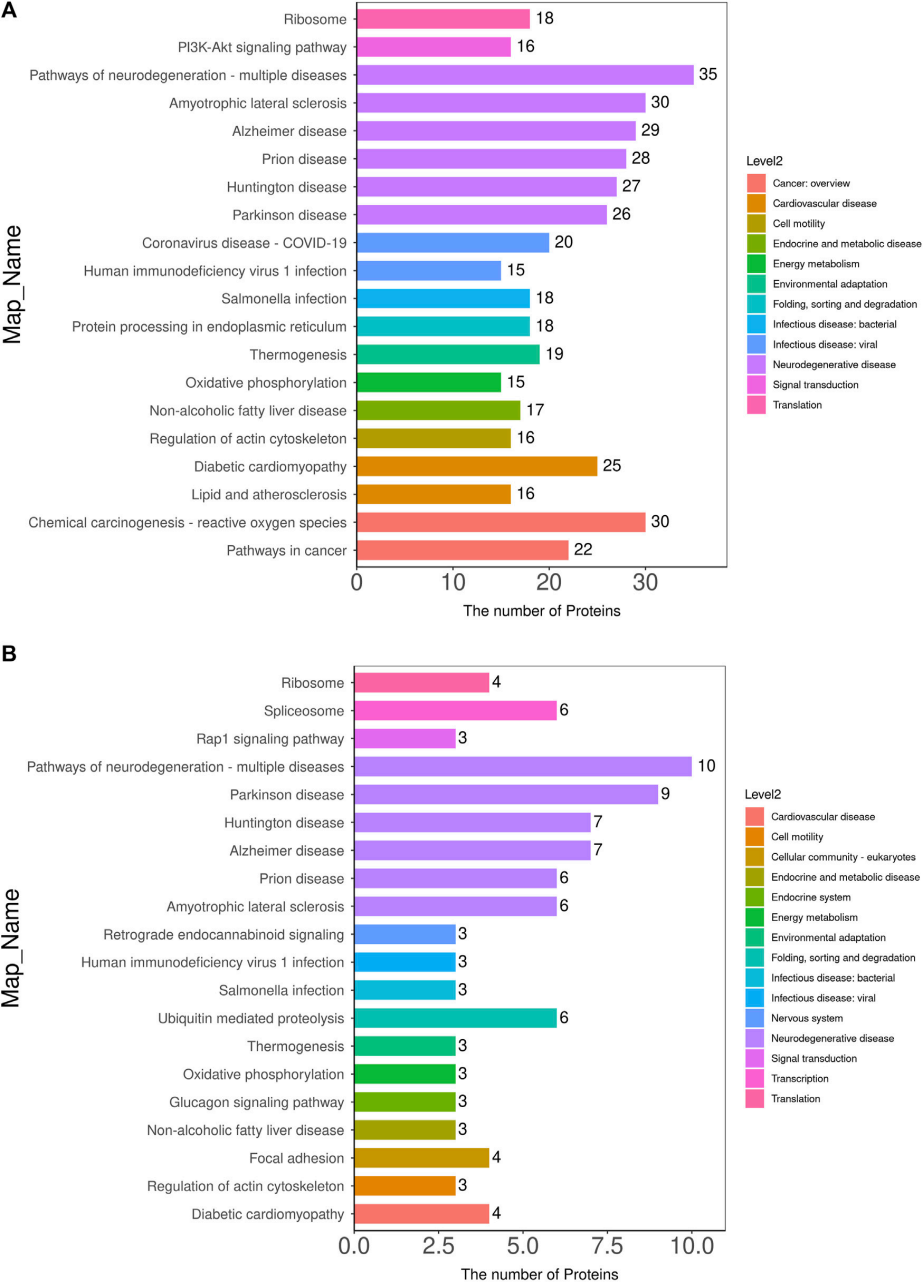
KEGG pathway annotation and attribution histogram of differentially expressed proteins. KEGG pathway annotation of differentially expressed proteins between the MOD group and the NC group **(A)** and between the KR-EA-H group and the MOD group **(B)**. X-axis represents the number of proteins involved in each pathway, and the y-axis lists the specific KEGG pathway names. Different colors in the legend represent different pathway categories. Red indicates cancer, orange indicates nervous system disease, yellow indicates energy metabolism, green indicates digestive system disease, cyan indicates infectious disease, blue indicates signal transduction, purple indicates immune disease, pink indicates endocrine and metabolic disease, and brown indicates environmental adaptation.

**FIGURE 6:**
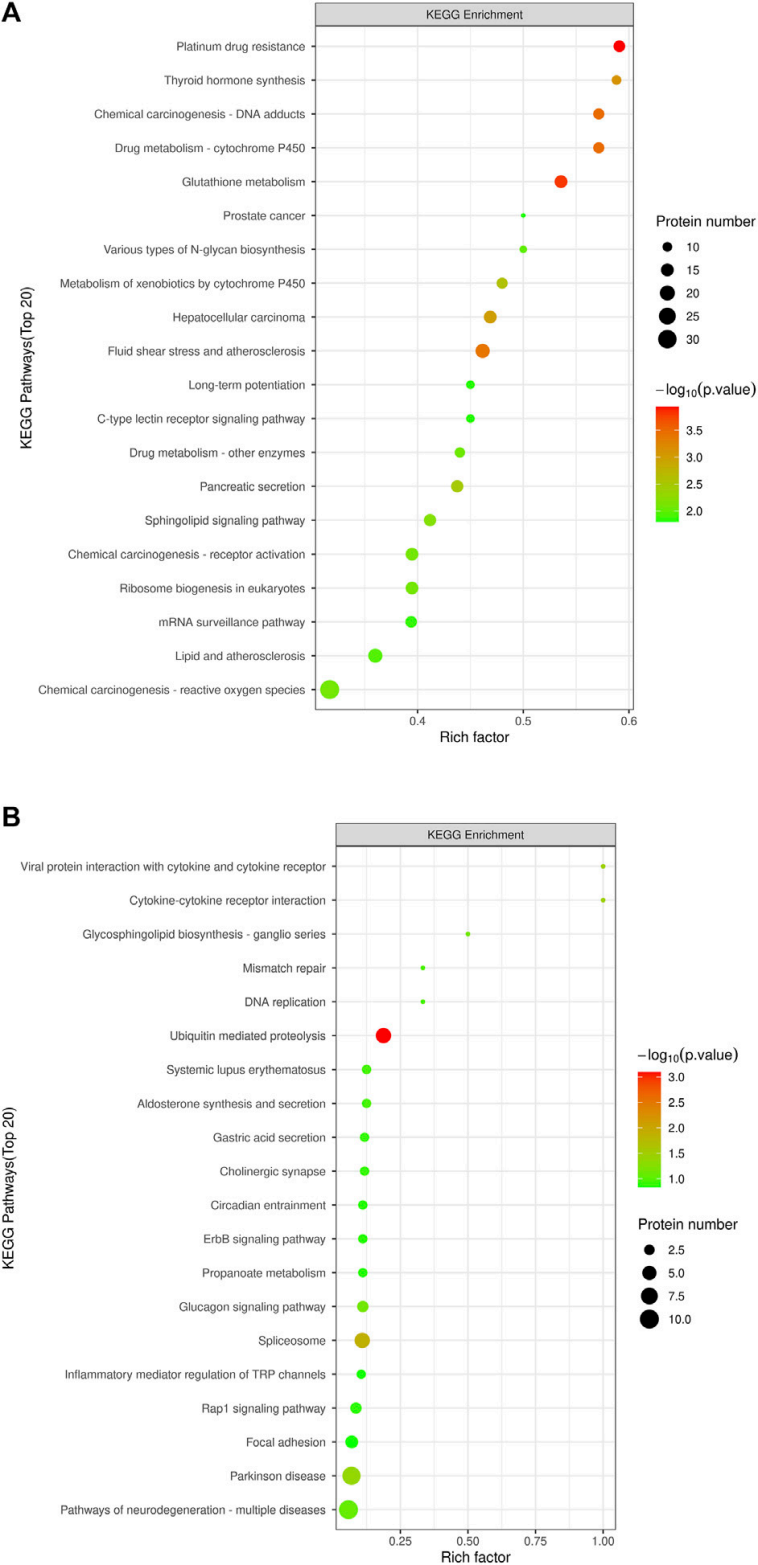
KEGG pathway enrichment bubble chart of differentially expressed proteins. The KEGG pathway enrichment plot shows the top 20 enriched pathways of differentially expressed proteins between the MOD group and the NC group **(A)** and between the KR-EA-H group and the MOD group **(B)**. X-axis Rich factor represents the ratio of the number of differentially expressed proteins observed in a specific pathway to the total number of proteins in that pathway, and y-axis lists the specific KEGG pathway names. The color of the bubbles represents the number of proteins, with darker colors indicating a higher number of proteins. The size of the bubbles represents −log_10_ (P-value), i.e., enrichment significance, with larger bubbles indicating higher significance.

KEGG pathway enrichment analyses were performed on DEPs using Fisher’s Exact Test to systematically evaluate the significance of enriched metabolic pathways and identify the most valuable KEGG metabolic pathways for research. The results were visualized as bubble plots to highlight the characteristics of overall metabolic pathway enrichment. The metabolic pathways significantly enriched in the MOD group compared to the NC group, in descending order of significance, included platinum drug resistance, thyroid hormone synthesis, drug metabolism-cytochrome P450, chemical carcinogenesis-DNA adducts, glutathione metabolism, various types of N-glycan biosynthesis, prostate cancer, metabolism of exogenous compounds by cytochrome P450, liver cancer, fluid shear stress and atherosclerosis, C-type lectin receptor signaling pathway, pancreatic secretion, and sphingolipid metabolism, among others (Figure 5A). Figure 6 depicts the KEGG pathways significantly enriched in the KR-EA groups compared to the MOD group. These pathways, ranked by significance, included spliceosome, bacterial invasion of epithelial cells, ubiquitin-mediated protein hydrolysis, glucagon signaling pathway, carbon metabolism, insulin signaling pathway, propionate metabolism, proteoglycans in cancer, ErbB signaling pathway, starch, and sucrose metabolism. These findings indicate the distinct and biologically significant metabolic pathways involved in the progression of diabetes and the therapeutic effects of KR-EA treatment, providing valuable insights for future research.

#### 3.3.4 PRM validation

To further elucidate the glucose-lowering mechanism of *K. roxburghii* in diabetic rats, KEGG pathways enriched in the KR-EA group relative to the MOD group were analyzed to identify diabetes-related pathways. Eight metabolic pathways were identified: spliceosome, ubiquitin-mediated protein hydrolysis, glucagon signaling pathway, carbon metabolism, insulin signaling pathway, propionate metabolism, ErbB signaling pathway, and starch and sucrose metabolism. Proteins involved in these pathways were screened among the DEPs using the criteria FC > 1.2 or <0.83 and *P* < 0.05. A total of 17 DEPs were identified and subjected to PRM validation. Six proteins were validated as being related to diabetes and consistent with the glucose-lowering effects observed in the KR-EA groups, as shown in Table 3. The PRM validation results of these six proteins were consistent with the trends observed in the proteomic analysis, confirming the reliability of the proteomic findings. These validated proteins are implicated in key metabolic pathways and play a critical role in the glucose-lowering effects of KR-EA.

**TABLE 3:** PRM validation results of differentially expressed proteins in diabetic rats treated with *Knoxia roxburghii* extract.

Protein ID	Gene ID	FC	PRM multiplier
ethylmalonyl-CoA decarboxylase	ECHDC1	0.76	0.53
calcium/calmodulin-dependent protein kinase II	CAMK2D	0.77	0.62
damage-specific	DDB1	0.80	0.65
ubiquitin-like modifier activati	UBA6	0.81	0.64
baculoviral IAP repeat-containi	BIRC6	0.62	0.68
Hexokinase 1	HK1	0.69	0.53

### 3.4 Metabolomics

#### 3.4.1 Significantly different metabolites

**FIGURE 7:**
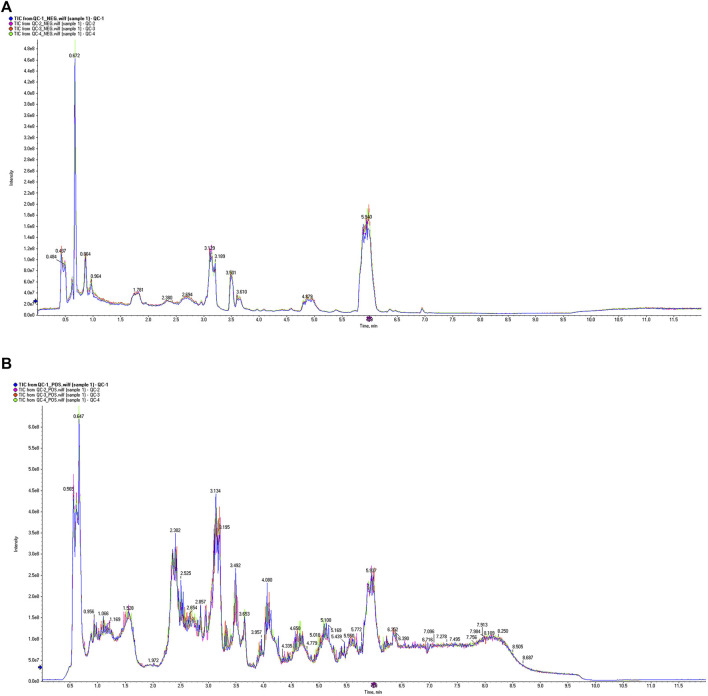
Total ion current diagram of the sample. Total ion current diagram in positive ion mode **(A)**, and Total ion current diagram in negative ion mode **(B)**. The different colors in the legend represent three replicate experiments of sample 1. The X-axis represents retention time, and the Y-axis represents the detected ion current intensity.

The VIP value, derived from the OPLS-DA model, can be used to measure the strength of the influence of the expression pattern of each metabolite on the taxonomic discrimination of each group of samples and to explain and uncover biologically significant differential metabolites. The total ion chromatograms (TIC) in positive and negative ionization modes are shown in Figure 7. Significant overlap of peak intensities and retention times across all chromatographic peaks in the figure indicates minimal instrumental variation throughout the experiment, confirming system stability and reliability. Metabolites with VIP >1 are considered significant contributors to the model. Using VIP >1 and *P* < 0.05 as screening criteria, 25 significant differential metabolites were identified in the positive ion mode and 40 in the negative ion mode in the MOD group compared with the NC group. In the KR-EA groups, three significant differential metabolites were identified in the positive ion mode compared with the MOD group: methylmalonic acid, arachidonic acid, and leucine. In the negative ion mode, 24 significant differential metabolites were identified, and the top ten metabolites were L-cysteine, linoleic acid, arachidonic acid (non-peroxidized), (Z)-9,12,13-trihydroxyoctadeca-15-enoic acid, succinic acid, meso-chlorohydroxyseafoamphonic acid, cis-4,7,10,13,16,19-docosahexaenoic acid, acetoacetic acid, methyl hexadecanoate, and L-sitosterol-1,4-lactone. Details of these metabolites are presented in Tables 4, 5. Not all metabolites were enriched in KEGG pathways and do not have KEGG code numbers in the tables.

**TABLE 4:** Screening and analysis of serum metabolites with significant differences in *Knoxia roxburghii* extract-treated diabetic rats (KR-EA-H vs. MOD).

Retention time(s)	ID	ESI	VIP	Potential biomarker	HMDB ID	FC	Change trend
132.3990	M353T132	+	1.18416517	15(r),19(r)-hydroxyprostaglandin f2.alpha.		0.56690452	↓
89.7100	M363T90	+	1.9196339	Hydrocortisone	HMDB0000063	1.69743558	↑
60.3500	M785T60	+	1.07474449	1-palmitoyl-2-stearoyl-sn-glycero-3-phosphocholine	HMDB0007970	1.24296664	↑
392.3165	M117T392	-	1.970400967	Methylmalonic acid	HMDB0000202	1.714513582	↑
49.9070	M311T50_2	-	1.466996227	Arachidic acid	HMDB0002212	1.35942313	↑
267.1145	M130T267	-	3.988962547	Leucine	HMDB0000687	1.264353304	↑
72.0500	M164T72	-	4.85768742	L-homocysteic acid	HMDB0002205	0.83142451	↓
50.5980	M279T51	-	14.4105483	Linoleic acid	HMDB0000673	0.75925572	↓
49.6320	M303T50	-	12.4631413	Arachidonic acid (peroxide free)	HMDB0001043	0.6910187	↓
167.4490	M329T167_2	-	2.29418408	(z)-9,12,13-trihydroxyoctadec-15-enoic acid		0.68460573	↓
107.6935	M117T108	-	1.0311032	Succinate	HMDB0000254	0.68015226	↓
196.4280	M213T196	-	1.68939175	m-Chlorohippuric acid		0.67110452	↓
51.2415	M327T51	-	5.5557162	Cis-4,7,10,13,16,19-docosahexaenoic acid	HMDB0002183	0.66280262	↓
308.1275	M101T308	-	1.91917397	Acetoacetic acid	HMDB0000060	0.63483884	↓
103.8665	M315T104	-	6.4283328	Methyl hexadecanoate	HMDB0061859	0.63306363	↓
135.8825	M177T136	-	5.01321611	L-gulono-1,4-lactone	HMDB0003466	0.63012468	↓
308.2795	M71T308	-	1.92225086	Pyruvaldehyde	HMDB0001167	0.6069025	↓
308.1795	M149T308	-	2.14198457	D-Lyxose		0.59767633	↓
308.1415	M179T308_2	-	15.2304229	D-Mannose	HMDB0062473	0.58515857	↓
51.8885	M277T52	-	4.3927977	Linolenic acid	HMDB0001388	0.5662968	↓
75.5405	M281T76	-	3.60118204	Oleic acid	HMDB0000207	0.55156162	↓
105.8105	M311T106	-	3.70881882	Fa 18:2+2o		0.5399724	↓
308.4220	M239T308	-	4.56788466	Alpha-D-Glucose	HMDB0003345/HMDB0061922	0.52597591	↓
42.5580	M144T43	-	1.59802692	Indole-3-carboxaldehyde	HMDB0029737	0.48214642	↓
308.6395	M359T309	-	4.30600136	D-fructose	HMDB0062538	0.47642663	↓
312.6150	M158T313	-	0.07520753			1.04762337	↓
308.6180	M299T309	-	1.28169067	Farrerol	HMDB0130571	0.47110549	↓
135.8290	M355T136	-	1.50580112	L-Gulonic gamma-lactone	HMDB0003466	0.44638659	↓

**TABLE 5:** Screening and analysis of serum metabolites with significant differences in *Knoxia roxburghii* extract-treated diabetic rats (MOD vs. NC).

Retention time(s)	ID	m/z	ESI	Potential biomarker	HMDB ID	Fold change	VIP
278.3465	M144T278	144.09981	+	Stachydrine	HMDB0004827	1.89823191	1.70115277
371.2625	M217T371	217.12732	+	Pro-thr		1.72806972	1.26041794
103.7855	M61T1042	61.03905	+	Urea	HMDB0000294	1.5272811	2.40114889
269.6185	M159T2702	159.11096	+	Val-Ala-Lys		1.38773776	1.3038898
146.9920	M773T147	772.58060	+	1,2-dioleoyl-sn-glycero-3-phosphoethanolamine-n,n-dimethyl	HMDB0010564	1.18991405	1.02307058
193.6425	M522T194	522.35323	-	1-oleoyl-sn-glycero-3-phosphocholine	HMDB0002815	0.84883359	2.34425599
196.6075	M496T197	496.34307	-	1-palmitoyl-sn-glycero-3-phosphocholine	HMDB0010382	0.83286319	8.74199663
176.4960	M814T176	813.68336	-	N-nervonoyl-d-erythro-sphingosylphosphorylcholine	HMDB0012107	0.76423038	3.50027019
149.9860	M735T150	734.56550	-	1,2-dihexadecanoyl-sn-glycero-3-phosphocholine	HMDB0000564	0.75883516	1.49815379
65.3845	M123T652	123.05357	-	Niacinamide	HMDB0001406	0.75015141	1.77185999
399.9420	M130T400	130.04771	-	Goitrin		0.71041635	1.19764673
143.6120	M769T144	768.58504	-	1-hexadecyl-2-(5z,8z,11z,14z-eicosatetraenoyl)-sn-glycero-3-phosphocholine		0.70009088	1.20394604
367.6080	M132T368	132.07475	-	Creatine	HMDB0000064	0.69487376	1.29547504
173.1820	M426T173	426.35479	-	Oleoyl-l-carnitine	HMDB0005065	0.69169732	1.12811116
208.2795	M455T208	455.18686	-	2'-deoxycytidine	HMDB0000014	0.68056279	3.16228089
262.3595	M232T262	232.15262	-	(r)-butyrylcarnitine	HMDB0002013	0.6237917	1.69050828
200.5455	M468T201	468.30536	-	1-myristoyl-sn-glycero-3-phosphocholine	HMDB0010379	0.60426216	1.36255114
62.7715	M302T63	302.30420	-	Pro-Trp	HMDB0000269	0.58332437	3.35864073
176.0575	M400T176	400.33948	-	L-palmitoylcarnitine	HMDB0000222	0.56466578	1.64513078
58.4885	M356T58	356.35007	-	Arachidoyl ethanolamide		0.55537514	1.39746516
71.7470	M246T722	246.24109		2,4,6-tri-tert-butylaniline		0.44673906	3.36637002
67.1985	M390T67	390.35496	-	N-octanoylsphingosine		0.37013434	1.20876041
60.3655	M400T60	400.37630	-	Demissidine		0.36133552	1.1376858
529.3065	M175T529	175.11714	-	Arginine	HMDB0000517	0.35060012	4.85366176
325.8440	M248T326	248.14701	-	3-hydroxybutyrylcarnitine	HMDB0062735	0.34596529	1.43552657
27.7300	M107T28	107.05202	-	4-methylphenol	HMDB0001858	3.32643493	3.01886828
113.3695	M116T113	116.05016	-	3-aminopentanoic acid		2.75469211	1.33767187
113.2030	M188T113	188.07133	-	1h-indole-3-propanoic acid	HMDB0002302	2.73323187	6.27848582
308.6395	M359T309	359.11701	-	D-fructose	HMDB0062538	2.68976496	3.22007886
135.8290	M355T136	355.08571	-	L-Gulonic gamma-lactone	HMDB0003466	2.61722075	1.16275869
106.9320	M331T107	331.18914	-	Prostaglandin e3	HMDB0002664	2.5991647	1.35509377
311.6795	M200T312	199.96828	-	Cysteine-s-sulfate	HMDB0000731	2.58621636	1.10556522
308.4220	M239T308	239.07594	-	Alpha-D-Glucose	HMDB0003345/HMDB0061922	2.40728381	3.47039221
30.9160	M212T31	212.00554	-	Indoxyl sulfate	HMDB0000682	2.30820659	10.7849811
308.1415	M179T3082	179.05693	-	D-Mannose	HMDB0062473	2.18924674	12.0626353
104.4350	M335T104	335.22029	-	Prostaglandin b1	HMDB0002982	2.15859722	2.1469429

#### 3.4.2 Significant enrichment analysis of the KEGG pathway

**FIGURE 8:**
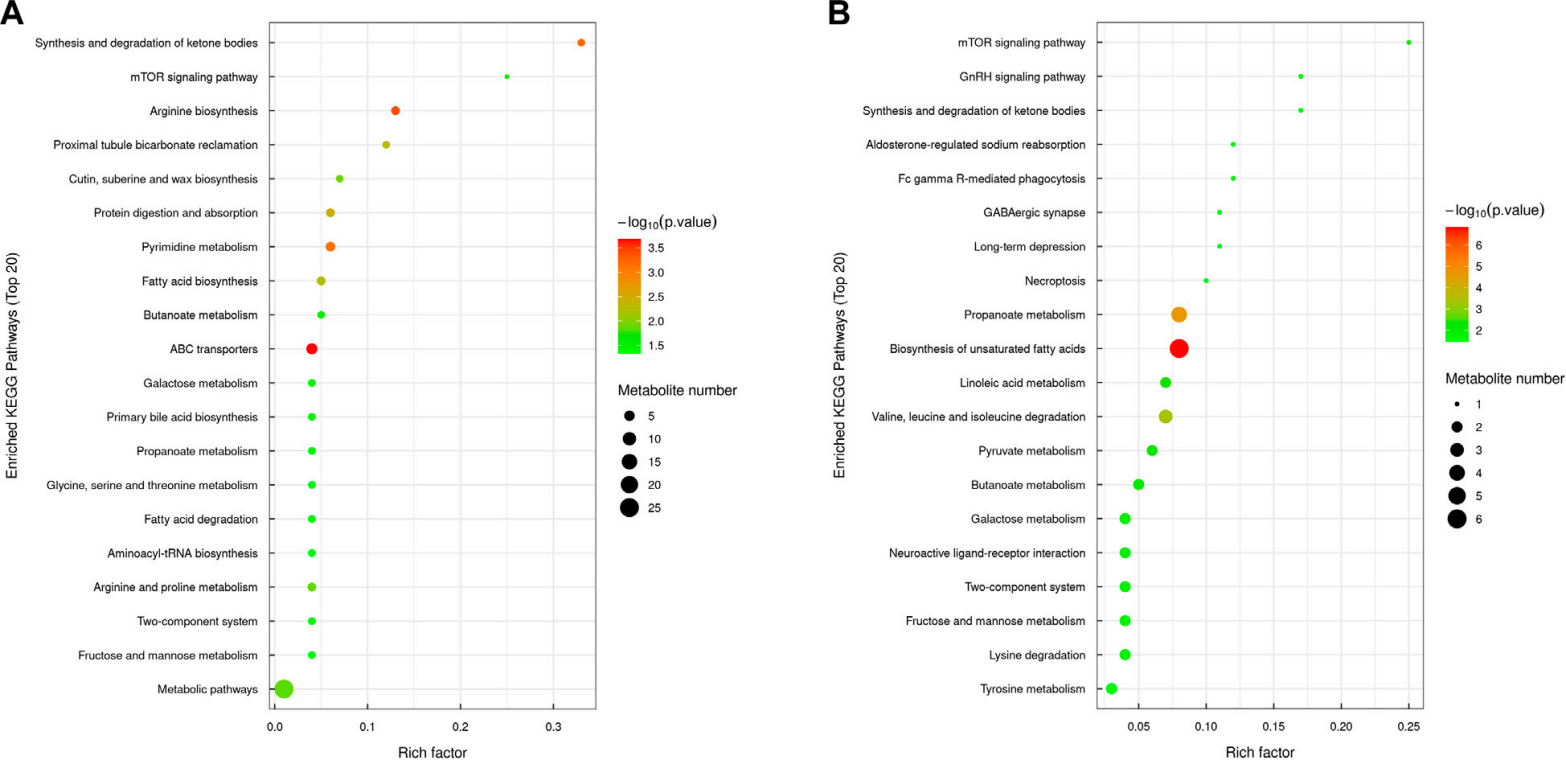
KEGG pathway enrichment bubble chart of differential metabolites. The KEGG pathway enrichment chart shows the top 20 pathways enriched with differentially expressed metabolites between the MOD group and the NC group **(A)** and between the KR-EA-H group and the MOD group **(B)**. X-axis Rich factor represents the ratio of the number of differentially expressed metabolites observed in a specific pathway to the total number of metabolites in that pathway, and y-axis lists the specific KEGG pathway names. The color of the bubbles represents the number of metabolites, with darker colors indicating a higher number of metabolites. The size of the bubbles represents −log_10_ (P-value), i.e., enrichment significance, with larger bubbles indicating higher significance.

The significant metabolic pathways enriched in the MOD group compared with the NC group included arginine biosynthesis, ketone body synthesis and degradation, protein digestion and absorption, pyrimidine metabolism, butyric acid metabolism, propionate metabolism, ABC transporter, fatty acid biosynthesis and degradation, fructose and mannitol metabolism, glycine, serine, and threonine metabolism, bile acid biosynthesis, galactose metabolism, and the mTOR signaling pathway. In the KR-EA group compared with the MOD group, the significantly enriched pathways included unsaturated fatty acid biosynthesis, the mTOR signaling pathway, propionate metabolism, linoleic acid metabolism, ketone body synthesis and degradation, valine, leucine, and isoleucine metabolism, galactose metabolism, pyruvate metabolism, butyric acid metabolism, fructose and mannitol metabolism, and the GnRH signaling pathway. A comparison of both groups revealed six commonly involved pathways: butyric acid metabolism, propionate metabolism, the mTOR signaling pathway, fructose and mannitol metabolism, galactose metabolism, and ketone body synthesis and degradation. These shared pathways suggest that KR-EA may exert its therapeutic effects on diabetes mellitus by regulating these critical metabolic processes (Figure 8).

## 4 Discussion and summary

Diabetes mellitus is a multifactorial metabolic disorder characterized by chronic hyperglycemia and its associated complications, including organ damage, oxidative stress, and endocrine dysfunction. Extensive research on TCMs and their formulations has demonstrated significant therapeutic potential in diabetic animal models. Studies indicate that TCMs effectively lower blood glucose levels by increasing insulin secretion, improving insulin resistance, modulating glucose and lipid metabolism, and mitigating oxidative stress (Lin and Zhang, 2023; Su et al., 2023; Zhang et al., 2020; Zhao et al., 2021). In this study, a diabetic rat model was established via intraperitoneal injection of STZ, a compound derived from *Streptomyces achromogenes* that is widely employed in rodent models of diabetes (Yan and Wu 2015). STZ selectively destroys pancreatic β-cells by inducing DNA methylation, impairing insulin production, and persistent hyperglycemia. Due to its transient effects, the resultant hyperglycemia is primarily attributed to temporary glucose toxicity, making STZ a commonly used agent for establishing type 1 and type 2 diabetes models (Furman, 2021; Goyal et al., 2016). Current protocols typically employ high-dose STZ injections to model type 1 diabetes and combine high-fat diet feeding with low-dose STZ to induce type 2 diabetes. Based on preliminary findings, a dose of 40 mg/kg STZ was selected in this study to induce diabetes, with no distinction made between diabetes subtypes. Following successful model induction, rats in the model group showed significantly elevated blood glucose levels compared with the normal group, along with classic symptoms of “three excesses and one deficiency” (polydipsia, polyphagia, polyuria, and weight loss) throughout the experimental period. Upon treatment with *K. roxburghii*, FBG levels were significantly reduced, FINS levels increased, and both islet morphology and area improved in diabetic rats. To further elucidate the potential mechanism of *K. roxburghii* in diabetes treatment, 4D data-independent acquisition (DIA)-based proteomics and serum metabolomics were conducted to identify DEPs and associated metabolic pathways. The 4D untargeted proteomics technique quantifies protein abundance via mass spectrometry coupled with temporal ion mobility information, allowing dynamic profiling of protein expression (Meier et al., 2020). Proteomic analysis revealed eight significantly enriched pathways in the treatment group relative to the model group, including the spliceosome, ubiquitin-mediated proteolysis, glucagon signaling pathway, carbon metabolism, insulin signaling pathway, propanoate metabolism, ErbB signaling pathway, and starch and sucrose metabolism. The findings indicate that the ethyl acetate extract of *K. roxburghii* may exert its anti-diabetic effects by downregulating several key proteins, including ECHDC1, CAMK2D, DD B1, UBA6, BIRC6, and HK1.

At the level of glucose metabolism and insulin signaling pathways, HK1 functions as a key regulatory enzyme in glycolysis. While its activity typically facilitates glycolytic flux and glucose utilization, hyperglycemic conditions can induce excessive HK1 activity, promoting aberrant glucose flux and metabolic dysregulation ((Rabbani and Thornalley, 2023; Yousri et al., 2023). Elevated HK1 expression is associated with increased inflammatory cytokine production and oxidative stress, ultimately contributing to glucose metabolism dysregulation (Luo et al., 2022; Jin et al., 2022). In this study, HK1 activity in the treatment group was significantly lower than in the model group, indicating improved glucose homeostasis. CAMK2D is involved in insulin signaling and glucose regulation; its inhibition has been reported to increase insulin sensitivity, improve glucose tolerance, and reduce adipocyte inflammation (J. Chen et al., 2021; Dai et al., 2021; Ozcan et al., 2012; 2013). Inhibition of CAMK2D disrupts glucagon receptor-mediated cAMP signaling and inhibits glucagon/cAMP-induced glycogenolysis and gluconeogenesis, resulting in reduced blood glucose levels (Chen et al., 2021; Ozcan et al., 2012). ECHDC1 is implicated in mitochondrial fatty acid β-oxidation, catalyzing the decarboxylation of ethylmalonyl-CoA to butyryl-CoA and CO_2_ (Linster et al., 2011). ECHDC1 downregulation inhibits β-oxidation, reducing the substrate supply required for gluconeogenesis. Since excessive gluconeogenic activity contributes to hyperglycemia, downregulation of both CAMK2D and ECHDC1 reduces endogenous glucose production, improves insulin receptor signaling, and alleviates hyperglycemia. All three proteins are involved to varying degrees in the processes of glycolysis and gluconeogenesis.

Under oxidative stress conditions, CAMK2D inhibition suppresses the NF-κB signaling pathway, reducing pro-inflammatory cytokine secretion (e.g., TNF-α, IL-6) and improving pancreatic β-cell function (Chen et al., 2025). BIRC6 is also involved in NF-κB pathway activation; thus, the co-downregulation of CAMK2D and BIRC6 may synergistically inhibit inflammatory signaling, interrupting the oxidative stress and inflammation cycle. Furthermore, downregulation of UBA6 decreases aberrant ubiquitination, reducing proteasomal overload and promoting autophagic clearance of damaged mitochondria (Jia and Bonifacino, 2020; 2019a). Simultaneous suppression of BIRC6 supports the removal of severely damaged cells via apoptosis, limiting the spread of reactive oxygen species (ROS) (Ehrmann et al., 2023; Zhang et al., 2020). Hyperactivation of the BIRC6/UBA6 axis impairs autophagy and promotes protein aggregation, leading to disrupted cellular homeostasis (Jia and Bonifacino, 2021; 2020; 2019b). DDB1 acts as a scaffolding protein for the E3 ubiquitin ligase complex and synergizes with UBA6 to promote CHIP-mediated ubiquitination of Nrf2 (Angers et al., 2006; Jin et al., 2006; 2006). Down of DDB1 reduces proteasomal degradation of Nrf2 (extending its half-life by more than threefold), thereby increasing β-cell survival. The stabilized Nrf2 concurrently upregulates SOD expression. (Li et al., 2022). Upregulation of SOD2 neutralizes mitochondrial superoxide, thereby alleviating ER stress to protect β-cells from apoptosis (Newsholme et al., 2019). Morphological improvements in pancreatic islets, including increased cellular volume and a higher nucleus-to-cytoplasm ratio, support restored insulin synthesis and β-cell function, highlighting *K*. *roxburghii’*s potential as a multi-target therapy against diabetes-associated oxidative damage.

While this study provides valuable insights into the therapeutic effects of *K. roxburghii*, several limitations warrant consideration. First, the active components responsible for the therapeutic effects of *K. roxburghii* were not identified. The analyses primarily focused on insulin secretion and oxidative stress; however, pancreatic islets consist of multiple functional cell types, including α-cells, β-cells, δ-cells, and PP-cells. A comprehensive evaluation of the effects of *K. roxburghii* on the interactions and functions of these cell types is essential. For instance, the hyperactivity of α-cells exacerbates (El and Campbell, 2020; Kim et al., 2017), while modulation of δ-cells could provide additional glycemic control (Huising et al., 2018). Second, the integration of proteomic and metabolomic data in this study was limited, restricting the identification of high-value therapeutic targets. Future research should adopt a multi-omics approach that integrates transcriptomics, proteomics, and metabolomics to construct a comprehensive molecular map of *K. roxburghii*’s therapeutic effects. Advanced techniques such as weighted gene co-expression network analysis (WGCNA) could further facilitate the identification of critical genes and pathways. Lastly, isolating and validating the active components of *K. roxburghii* through advanced analytical techniques such as LC-MS/MS and nuclear magnetic resonance (NMR) spectroscopy is necessary. Pharmacokinetic and bioavailability studies of these components will further improve their translational potential.

These findings indicate that *K. roxburghii* administration induced significant changes in glucose metabolism, insulin signaling, and oxidative stress responses, reflecting the multi-component, multi-target, and multi-pathway nature of traditional Chinese medicine. The extract suppressed gluconeogenesis, reduced oxidative damage in pancreatic cells, preserved islet structure, and promoted insulin secretion, contributing to lowering blood glucose levels and improving diabetic symptoms. The findings validate the therapeutic efficacy of *K. roxburghii* in diabetes management, which helps explain why physicians in Southwest China clinically employ this herb for diabetes treatment. Future studies focusing on the isolation and identification of active compounds from *K. roxburghii* may aid in developing novel therapeutic agents for diabetes. Given its endangered status, enhanced conservation of wild *K. roxburghii* resources, expansion of cultivated plantations, and accelerated pharmacological research on its active constituents are imperative.

## Data Availability

The original contributions presented in the study are included in the article/Supplementary Material, further inquiries can be directed to the corresponding authors.

## Glossary

AGC: Automatic Gain Control
AUC: Area under the Curve
BP: Biological Process
BIRC6: Baculoviral IAP Repeat - Containing Protein6
CAMK2D: Calcium/Calmodulin - Dependent Protein Kinase II Delta
CC: Cellular Component
DDB1: DNA Damage - Binding Protein 1
DEPs: Differentially Expressed Proteins
DMSO: Dimethyl sulfoxide
EA: Ethyl Acetate
ECHDC1: Ethylmalonyl - CoA Decarboxylase 1
ESI: Electrospray Ionization
FC: Fold Change
FASP: Filter - Assisted Sample Preparation
FINS: Fasting Insulin
GO: Gene Ontology
GSIS: Glucose - Stimulated Insulin Secretion
H&E: Hematoxylin and Eosin
HK1: Hexokinase 1
HILIC: Hydrophilic Interaction Liquid Chromatography
HOMA - β: Homeostasis Model Assessment - β - cell function
IDA: Information - Dependent Acquisition
INS: Insulin
KEGG: Kyoto Encyclopedia of Genes and Genomes
KR: *Knoxia roxburghii*
KR - EA - H: High - dose *Knoxia roxburghii* Ethyl Acetate Extract Group
KR - EA - L: Low - dose *Knoxia roxburghii* Ethyl Acetate Extract Group
KR - EA - M: Medium - dose *Knoxia roxburghii* Ethyl Acetate Extract Group
LC - MS/MS: Liquid Chromatography - Tandem Mass Spectrometry
MDA: Malondialdehyde
MF: Molecular Function
MS: Mass Spectrometry
NMR: Nuclear Magnetic Resonance
OGTT: Oral Glucose Tolerance Test
OPLS - DA: Orthogonal Partial Least Squares Discriminant Analysis
PCA: Principal Component Analysis
PRM: Parallel Reaction Monitoring
PRTC: Peptide Retention Time Calibration
SD: Sprague - Dawley
SDT: Sodium Dodecyl Sulfate - Tris - EDTA Buffer
SOD: Superoxide Dismutase
STZ: Streptozotocin
SVR: Support Vector Regression
TCMs: Traditional Chinese Medicines
TIC: Total ion chromatograms
TP: Total Protein
UHPLC: Ultra - High - Performance Liquid Chromatography
UBA6: Ubiquitin-Like Modifier-Activating Enzyme 6
VIP: Variable Importance for Projection
WGCNA: Weighted Gene Co - expression Network Analysis
